# Is sweat testing for cystic fibrosis feasible in patients with down syndrome?

**DOI:** 10.1186/s12890-018-0580-1

**Published:** 2018-01-16

**Authors:** Katharina Ruf, Antonia Demerath, Helge Hebestreit, Steffen Kunzmann

**Affiliations:** 1 University Children’s Hospital, Würzburg, Germany; 2 Clinic of Neonatology and Pediatric Intensive Care, Bürgerhospital Frankfurt am Main, Frankfurt am Main, Germany; 3 Children’s Hospital of the University of Würzburg, Josef-Schneider Str. 2, 97080 Würzburg, Germany

**Keywords:** Sweat secretion rate, Sweat osmolality, Gender gap, Non-responder, Thermoregulation

## Abstract

**Background:**

Recurrent airway infections are common in patients with Down’s syndrome (DS). Hence, ruling out Cystic Fibrosis (CF) in these patients is often required. In the past, the value of sweat testing – the gold standard to diagnose CF – has been questioned in DS as false positive results have been reported. However, these reports are based on measurements of sweat osmolality or sodium concentrations, not chloride concentrations. This study analyses sweat secretion rate and chloride concentration in sweat samples of patients with DS in comparison to healthy controls.

**Methods:**

We assessed sweat samples in 16 patients with DS and 16 healthy controls regarding sweat secretion rate (SSR) and sweat chloride concentration.

**Results:**

All measured chloride concentrations were within the normal range. The chloride concentrations were slightly, but not significantly lower in patients with DS (15,54 mmol/l (±4,47)) compared to healthy controls (18,31 mmol/l (±10,12)). While no gender gap in chloride concentration could be found, chloride concentration increased with age in both groups.

Insufficient sweat was collected in 2 females with DS (12.5% of the study group) but not in an individual of the control group. A significant lower sweat secretion rate was found in the DS group (27,6 μl/30 min (± 12,18)) compared to the control group (42,7 μl/30 min (± 21,22)). In a sub-analysis, female patients produced significantly less sweat (20,8 ± 10,6 μl/30 min) than male patients with DS (36,4 ± 7,8 μl/30 min), which accounts for the difference between patients and controls. Furthermore, while the sweating secretion rate increased with age in the control group, it did not do so in the DS group. Once again this was due to female patients with DS, who did not show a significant increase of sweat secretion rate with age.

**Conclusions:**

Sweat chloride concentrations were within the normal range in patients with DS and therefore seem to be a reliable tool for testing for CF in these patients. Interestingly, we found a reduced sweat secretion rate in the DS group. Whether the last one has a functional and clinical counterpart, possibly due to a disturbed thermoregulation in DS patients, requires further investigation.

## Background

In patients with Down’s syndrome (DS), infections, especially of the airways, are more common than in the healthy population [1]. Children with frequent infections of the lower airways, though, require assessments to rule out cystic fibrosis (CF). Furthermore, as in patients with CF, children with DS are small of stature [2]. However, only few and old references of results and validity of sweat tests in these children exist [3–6].

An association of DS and CF has been described in the past and is rare but existent and case reports have been published [3–6]. The expected incidence of both conditions co-existing is about 1:2,650,000 in the United Kingdom (UK) (incidence of CF 1:2415, DS 1:1100 live births) [3]. The documented outcome of the combined disease is normally extremely poor, but also moderate courses have been described [4, 5].

An early diagnosis of CF, especially in co-existence with other chronic diseases like DS is crucial for therapy and therefore a reliable diagnostic tool is needed. The gold standard for diagnosing CF is the sweat test [7]; however, it is unclear, how reliable that tool is for children with DS. Changes in sweat gland anatomy have been described in DS, which could result in alterations of sweating rate and/or sweat electrolyte concentration [8].

In the past, Symon et al. and Geetha et al. reported an elevated sweat osmolality in children with DS who did not show any signs for CF [9, 10]. In contrast, Chapman et al. reported normal sweat sodium in 13 subjects with DS [11]. Milunsky et al. published three children with DS and CF diagnosed by increased concentration of sweat sodium and chloride [4] and Vetrella et al. described a child with DS and CF with raised sweat sodium and chloride concentration [6]. The gold standard for the diagnosis of CF nowadays is the measurement of sweat chloride concentration and not sweat osmolality after pilocarpine ionthophoreses [7].

To our knowledge, data on sweat chloride concentration in people with DS but no signs of CF does not yet exist. Therefore, we aimed to assess such concentrations. Based on the existing literature, we hypothesised that sweat chloride concentrations would be elevated in DS.

## Methods

### Study population

The study was approved by the local ethics committee (votum number 17/10; 03.03.2010). 16 patients with DS and 16 healthy controls were included in the study. They were recruited through paediatricians and self-help groups. Written informed consent was obtained from the patients and their legal guardians, if appropriate. Genotyping was not done in this population.

### Sweat test

The participants of the study were first familiarized with the equipment. In each participant, sweat collection was performed in duplicate, once on the left and once on the right arm. Pilocarpine-Iontophoresis was performed for 5 min (Wescor Modell 3700; Kreienbaum Neoscience GmbH, Langenfeld, Germany) [12]. Afterwards sweat was collected using a Macroduct Sweat Collection device for 30 min (Wescor Modell 3700; Kreienbaum Neoscience GmbH, Langenfeld, Germany) on each arm. The sweat secretion rate (SSR) was measured. Afterwards sweat chloride concentration (mmol/l) was analysed by using the ChloroChek® Chloridometer® (Kreienbaum Neoscience GmbH, Langenfeld, Germany), if the sweat amount was above 15 μl. If sweat amount was below 15 μl/30 min on both arms, the person was regarded as non-responder [13].

According to current European guidelines, a sweat test is considered indicative for CF, if the chloride concentration is above 60 mmol/l, a test is interpreted as intermediate if the chloride concentration ranges between 30 and 59 mmol/l. CF is unlikely if the chloride concentration remains below 30 mmol/l [7].

### Statistical analysis

Statistical analyses were performed by using SPSS Statistics 23 (IBM). Differences between patients with DS and controls as well as between male and female participants were calculated by using Student’s t-test for normally distributed data and Mann-Whitney U test, if data was not normally distributed. For correlations of age and sweat secretion rates, Spearmoan-Rho-correlations were calculated.

## Results

### Study population

The participants´ characteristics can be seen in Table 1. Both the DS group and the control group contained children and adults. Except for cardiac defects (10 in the DS group (56%), none in the control group (0%)) and hypothyreosis (7 in the DS group (43%), none in the control group (0%)), no difference was found with regard to concomitant diseases (see Table 2). All patients with DS and hypothyreosis were treated with thyroid hormones; as no blood was drawn in this study, levels of thyroid hormones are not available to show whether the replacement therapy was sufficient. No person in the DS and control group showed clinical signs for CF (respiratory or gastrointestinal symptoms), no participant was genotyped.

**Table 1:** Participants’ characteristics

	Patients with DS	Healthy controls
Number	16	16
sex	7 male	6 male
	9 female	10 female
Age (mean/range)	14,4 (3–32)	15,9 (3–30)
Age < 18 years	11	7
Weight (kg)	41,7 (18–64)	47,5 (17–81) ^n.s.^
Height (cm)	140 (99–164)	154 (103–182) ^n.s.^
Children with height below 3rd percentile	7/11	0/7*
Cardiac defects	10	0*
Hypothyreosis	7	0*

Comparison of the participants’ anthropometric data as well as concomitant illnesses. Significant differences are marked as follows: n.s. = non significant; * *p* < 0.05.

**Table 2:** Concomitant medication

Concomitant Medication	Patients with DS	Healthy controls
L-Thyroxine	7/16	1/16
MAO-Inhibitor	0/16	1/16
Birth control pills	1/16	3/16
Insulin	1/16	0/16
Methylphenidate	0/16	1/16
Inhalative Budesonide	0/16	1/16

Concomitant medication of the participants

As Cystic Fibrosis newborn screening via IRT-screening has not been regularly available until October 2016 in Germany, none of the participants underwent this screening, therefore we cannot report on such data.

### Sweat chloride concentration

**Fig. 1:**
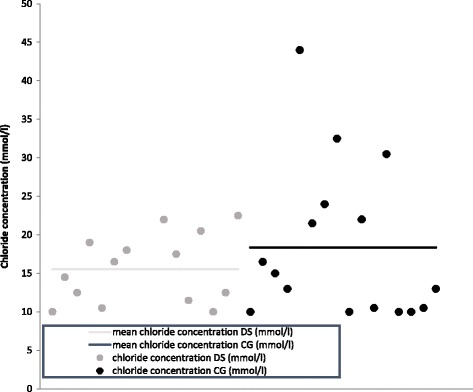
Differences in chloride concentration (mmol/l) between the DS and CF group

We analysed the chloride concentration in the sweat test samples in our participants. All measured chloride concentrations in both groups showed values clearly below 60 mmol/l (Fig. 1). Thus, none of the tests was indicative for CF. In our population, the mean chloride concentration was 15,54 mmol/l (± 4,47) in patients with DS and 18,31 mmol/l (± 10,12) in healthy controls. This difference proved not to be statistically significant.

#### Sweat chloride concentration in relation to sex

Sex did not have any influence on sweat chloride concentration, neither in the control group nor in the DS group (data not shown).

#### Sweat chloride concentration in relation to age

**Fig. 2:**
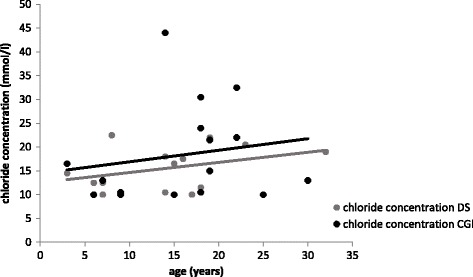
Increase of chloride concentration (mmol/l) with age in both groups

In both groups, patients with DS and healthy controls, sweat chloride concentration increased with age (Fig. 2). Also, no differences in the chloride concentration in relation to age were found between the both groups.

### Sweat secretion rate (SSR)

**Fig. 3:**
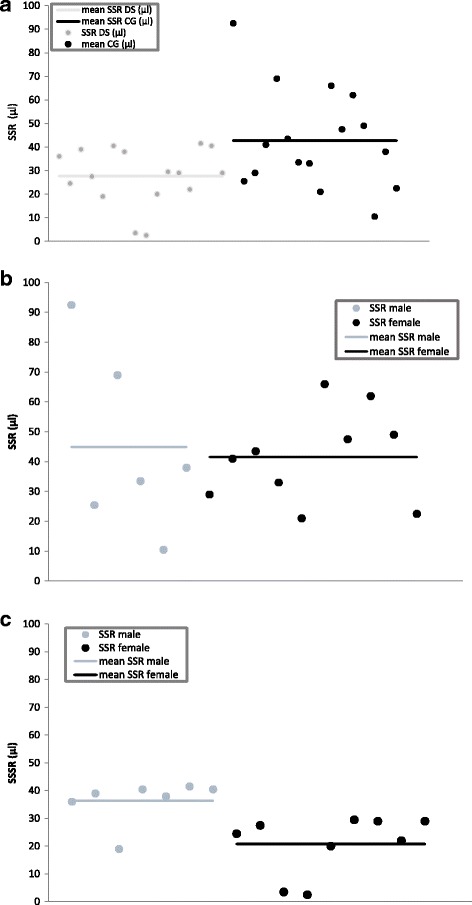
**a** Differences in sweat secretion rate (μl) between the DS and the CG participants. Participants of the DS group produced significantly less sweat (*p* < 0.05). **b** Sweat secretion rate (μl) of male and female participants of the CG. No difference in sweat secretion rate was observed. **c:** Significant difference in sweat secretion rate of male and female participants of the DS group. Females produced significantly less sweat (*p* < 0.05)

In the DS group, two of the 16 patients (both females) had a sweat secretion rate below 15 μl/30 min and had, thus, to be excluded from the analysis. In the control group, all participants produced enough sweat to meet the criterion for a valid test. In addition, there was a significant difference in the SSR between the two groups. The mean sweat amount after 30 min of collection was 27,6 μl (± 12,18) in the DS group and 42,7 μl (± 21,22) in controls (*p* < 0,05) (Fig. 3a).

#### SSR in relation to sex

When analysing the amount of sweat with regard to sex, it was evident that in the control group, the SSR was more or less equal in male (44,8 ± 30,3 μl) and female participants (41,5 ± 15,4 μl) (Fig. 3b). The patients with DS, however, showed a striking difference: female patients produced significantly less sweat (20,8 ± 10,6 μl) than male patients (36,4 ± 7,8 μl), which accounts for the difference between patients and controls (*p* < 0.05, see Fig. 3c). Of these female patients, 3 were pre- and 6 post-puberty.

#### SSR in relation to age

**Fig. 4:**
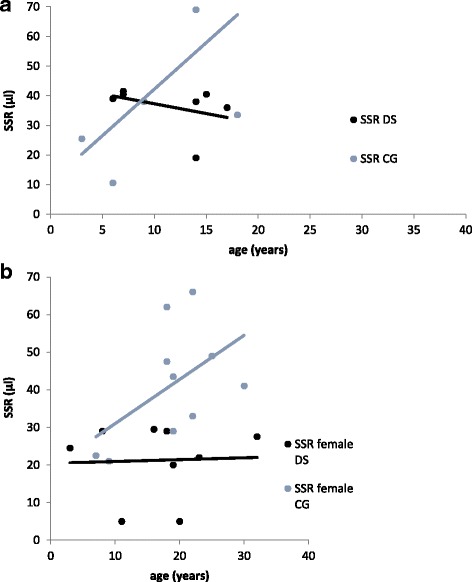
**a** Changes in the sweat secretion rate in the DS and the CG group. In the DS group, SRR does not increase with age. **b** Subanalysis of the changes in the sweat secretion rate of females in the DS and the CG group. In the DS group, SRR does not increase with age

While the SSR did not increase with age in patients with DS it did so in healthy controls (Fig. 4a). However, male participants in both groups were all younger than 20 years. In a sub-analysis of the females, though, it was the females of the DS group, who did not show a significant increase of SSR with age. Curves in Fig. 4 B represent the estimated female age-specific median for SSR in the DS and the control group.

## Discussion

Altered sweat tests have been reported for patients with DS for a long time, especially as the analysis always referred to sweat osmolality [8–10]. Up until now, no data on the measurement of chloride concentration in the sweat of patients with DS without CF has been published. Our data suggests that analysing sweat chloride concentration is a reliable tool to rule out CF in patients with DS, as we did not find any false positive results in our study population. However, this was a pilot study with only a small number of patients.

Some of the data generated on sweat tests in patients with DS have been generated by using the sweat osmolality as a surrogate marker for the sum of sweat electrolytes, especially potassium and sodium. It has been reported that the sweat osmolality is elevated in patients with DS without CF, leading to a false positive results in sweat tests for screening for CF in DS patients [10]. As there are too many influences on osmolality (i.e. hormones, age, sex, concomitant diseases), the analysis of the sweat chloride concentration has become the gold standard in diagnosing CF today [7]. In our study, the sweat amount of the participants was unfortunately too little to also measure sweat osmolality to confirm increased serum osmolality in the DS group, as described before [8, 9]. In contrast, the measured chloride and sodium (data for sweat sodium concentration was not shown, because of too little sweat amount in many participants) concentration in our study was even slightly, but not significant lower in patients with DS compared with the control group. In line with our results, Chapman et al. reported that in 13 children with DS, sweat sodium concentration was normal and not elevated [11]. Therefore, the electrolyte pattern for chloride and sodium seems not to be responsible for the increased sweat osmolality reported in DS.

In a sub-analysis, we further analysed the possible influence of sex and age on sweat chloride concentration in the two groups. While we found no differences between the two groups in relation to sex, the chloride concentration increased with age in both groups. The latter observation is consistent with previous reports, which described a tendency for the sweat chloride to decrease during the first year of life (this age was not included in our study) and then to increase with subsequent aging [14, 15]. In a study of more than 10.000 sweat tests, Traeger et al. also reported about a slightly higher sweat chloride concentration in females compared to males [16], a finding that was also confirmed by Mishra et al. [15]. This small gender gap could not be represented in our study, probably due to the small study population.

Besides chloride concentration, we studied possible differences in the non-responder rate and the SSR between the two groups. We recognized a higher non-responder rate and lower SSR in patient with DS in our study. A higher non-responder rate to iontophoresis in patients with DS has been described before in the literature (10% in DS group to 3% in normal population) [9]. Also, in the study of Symon et al., 20% (5/25) of patients with DS failed to produce sufficient sweat for osmometry [9]. This could be further confirmed in a study of Geetha et al., in which the proportion of non-responders to pilocarpine was significantly higher in DS patients (> 10%) then in the normal population (3%) [8]. The higher non-responder rate and the difficulty to obtain enough sweat could be due to an abnormal skin commonly found in children with DS [17]. In this context, the anatomy of sweat glands seems to be different in patients with DS. It has been described that the sweat coil size and the ratio of coil volume to duct length are abnormally small in DS patients [18, 19], although it is unclear whether this has any clinical correlate.

In a sub-analysis related to sex, we could show that female patients produced significantly less sweat compared to male patients with DS, which accounts for the difference in SSR between patients and controls. This gender gap could not be found in the control group. This sex difference in SSR specific for DS was not described before. However, a general sex difference of sweat secretion pattern in children with lower median values for SSR for girls than boys has been reported before [16, 20, 21]. In contrast, Rees et al. also found an increased SSR in men than in women, although this difference did not occur in pre-pubertal boys and girls [22].

In our small study, there was no difference in SSR between pre- and post-pubertal female DS patients. In general, hormone status significantly influences sweat physiology [8, 9]. Androgens as well as growth hormone (GH) seem to play a role here [21–23]. Children with GH deficiency show reduced SSR, while patients with acromegaly have a higher SSR than healthy controls [21, 23]. It is known that DS is associated with growth hormone deficiency [24]. In our albeit small population, children with DS and a height below the 3rd percentile showed no reduced SSR rate compared with the other children (data not shown). However, as we did not draw blood samples, GH levels could not be analysed. It remains mere speculation, whether lower GH levels are responsible for differences in SSR.

Another interesting and new finding was that the SSR did not increase with age in patients with DS, as it did in our control group and in other studies before [16, 25]. The different anatomy of sweat glands in DS, as mentioned before, could be a reason for this difference. When having a closer look, it is especially the female DS patients that account for this difference. Possibly, altered androgens in DS are responsible for this effect. If in a larger population the lower SSR in female patients with DS is confirmed, this may have clinical implications for patients with DS as thermoregulation may not function as well as in patients with a normal SSR. In general, sweating plays an important role for the maintenance of body temperature during exercise or in hot environments [26]. Accordingly, patients lacking sweat glands like in anhidrotic ectodermal dysplasia or patients with impaired sweating like in GH deficiency may be at risk for developing hyperthermia as a consequence of their decreased ability to sweat [27–30]. Especially conditions like fever and physical activity may put these DS patients at risk for hyperthermia and electrolyte derailment. It is certainly worthwhile to analyse this finding and its effects in a larger population.

### Limitations

All our findings were generated in a small sample. As an analysis of validity of chloride concentration in sweat tests in patients with DS has not happened so far, we started with this pilot study in order to have a proof of concept. We were able, though, to cover children as well as adults and male as well as female participants in both groups. Furthermore, we were able to compare patients with DS to healthy controls (without any recurrent lung infections or other CF suspicious symptoms). Due to the small sample, data was analysed using t-Tests and Mann-Whitney-U Tests. In a larger sample univariate analyses of variance or mixed linear models might be worthwhile to evaluate effects of sex and age; in this study, due to the number of participants, the power would have been to weak.

Another limitation is the low amount of sweat that was available for analysis. As we did double analyses (iontophoresis on both arms, analysis, and then calculating means of the results), we only had half of the amount for each test. Therefore, an analysis of osmolality and further electrolytes was possible only in a small group of participants. In further studies, it would be worthwhile to have just one analysis but the double amount of sweat to generate more data (e.g. osmolality, sodium concentration, conductivity). Also, drawing blood samples and having information on hormone status would be worthwhile to further understand differences in SSR.

As no genotyping was done in this study or afterwards in these patients, we cannot speculate, whether changes in sweat chloride levels may have been due to a carrier status, as reported by Ooi et al. [31].

## Conclusions

To our knowledge, this is the first study that shows that measuring chloride concentration in patients with Down’s syndrome without CF is a valid tool to discriminate between patients with CF and those without. Up until now, only osmolality or sodium concentration has been published to analyse the sweat, which lead to difficulty in diagnosis [8–10]. Using chloride concentration though, enables health professionals to not have false-positive sweat test results in patients with DS and is a valid tool to exclude a CF in patients with DS, a knowledge which is of high clinical relevance.

Another interesting finding deserves further analysis; the altered SSR in female patients with DS needs to be confirmed and if this is the case, medical implications of an impaired sweat rate need to be assessed.
